# Vertical Transfer of Humoral Immunity against Nipah Virus: A Novel Evidence from Bangladesh

**DOI:** 10.3390/tropicalmed8010016

**Published:** 2022-12-27

**Authors:** Syed Moinuddin Satter, Arifa Nazneen, Wasik Rahman Aquib, Sharmin Sultana, Mohammed Ziaur Rahman, John D. Klena, Joel M. Montgomery, Tahmina Shirin

**Affiliations:** 1 Programme for Emerging Infections, Infectious Diseases Division, icddr,b, Dhaka 1212, Bangladesh; 2 Institute of Epidemiology, Disease Control & Research, 44 Mohakhali, Dhaka 1212, Bangladesh; 3 Viral Special Pathogens Branch, Division of High Consequence Pathogens and Pathology, Centers for Disease Control and Prevention, Atlanta, GA 30333, USA

**Keywords:** Nipah virus, NiV, Bangladesh, vertical transfer, immunity, novel

## Abstract

A major obstacle to in-depth investigation of the immune response against Nipah virus (NiV) infection is its rapid progression and high mortality rate. This paper described novel information on the vertical transfer of immune properties. In January 2020, a female aged below five years and her mother from Faridpur district of Bangladesh were infected. Both had a history of raw date palm sap consumption and were diagnosed as confirmed NiV cases. The daughter passed away, and the mother survived with significant residual neurological impairment. She conceived one and a half year later and was under thorough antenatal follow-up by the surveillance authority. A healthy male baby was born. As part of routine survivor follow-up, specimens were collected from the newborn and tested for NiV infection at the reference laboratory to exclude vertical transmission. Although testing negative for anti-Nipah IgM and PCR for NiV, a high titre of anti-Nipah IgG was observed. The transfer of humoral immunity against NiV from mother to neonate was confirmed for the first time. The article will serve as a reference for further exploration regarding NiV-specific antibodies that are transferred through the placenta, their potential to protect newborns, and how this may influence vaccine recommendations.

## 1. Introduction

The Nipah virus (NiV) is a zoonotic pathogen that belongs to risk group 4 [1,2,3]. Fruit bats from the genus Pteropus are its natural reservoir, and spillover to the human population usually occurs through a medium, i.e., infected animals or food items contaminated with the virus [1,3,4,5,6]. NiV, one of the fatal emerging pathogens of our time, was first detected in the Malaysia–Singapore region in 1998, which sparked a devastating pandemic [1]. Hence, the virus is named after Kampung Sungai Nipah in Malaysia [4]. In Bangladesh, the virus was first reported in 2001, and since then, the NiV has become endemic to this densely populated country, with confirmed cases reported almost every year [3,7,8,9]. An alarming feature regarding disease transmission was the evidence of person-to-person transmission [4,7,9,10,11,12]. The virus usually causes respiratory infection followed by infection of the central nervous system [1,13]. The clinical presentation of human Nipah infection includes sudden onset fever, cough, respiratory distress, altered mental status, convulsion, and coma [1,2,8,9]. According to the available scientific evidence, two out of three infected individuals died during the infection [1,8,9]. Till 2021, 321 cases of NiV infection has been detected and only 96 of them survived the initial period of infectivity [14]. There are 85 NiV infection survivors in Bangladesh as of July 2021, according to an unpublished article entitled “A 16-year story of tackling a global epidemic threat: Nipah surveillance in Bangladesh, 2006–2021”. Since its emergence, infection was confirmed through ELISA for IgM and IgG against NiV and RT-PCR to detect viral nucleic acid [1,4,5,8,9]. Considering its public health risk and the level of panic it created in the community, in 2006 Institute of Epidemiology Disease Control and Research (IEDCR) and icddr,b jointly introduced the national Nipah surveillance in Bangladesh, with technical support from US CDC, Atlanta [5,7]. This initiative enabled the authority to identify and confirm NiV infection at the earliest, facilitate the implementation of mitigation measures to prevent disease transmission, as well as to conduct routine follow up of NiV infection survivors. The purpose of survivor follow-up was to monitor changes in disease sequelae and progression of immunological characteristics and to detect events of relapse/recrudescence at the earliest.

Human NiV infection is known for its rapid progression and high fatality. Thus, exploring virus–human interaction from an immunological perspective has been challenging. The minimum available scientific evidence suggest the presence of NiV RNA in blood and throat/oro-pharyngeal swab samples during the course of infection as well as the development and persistence of humoral immunity (Anti Nipah IgM and IgG) [15,16]. Currently, no vaccine or therapeutics are available to prevent or treat this disease. Several potential vaccines are in the pipeline, and immunological evidence from NiV infection survivors lays the foundation for this effort [1,4].

During routine follow-up, novel evidence of the transfer of immune properties from a survivor to her offspring was identified. This article elaborates on this newly found data and its implications on our understanding of immunity against NiV infection, passive transfer of immune properties, and future disease prevention efforts, especially vaccine development.

## 2. Materials and Methods

### 2.1. Surveillance Strategy

#### 2.1.1. Sentinel Surveillance

**Figure 1:**
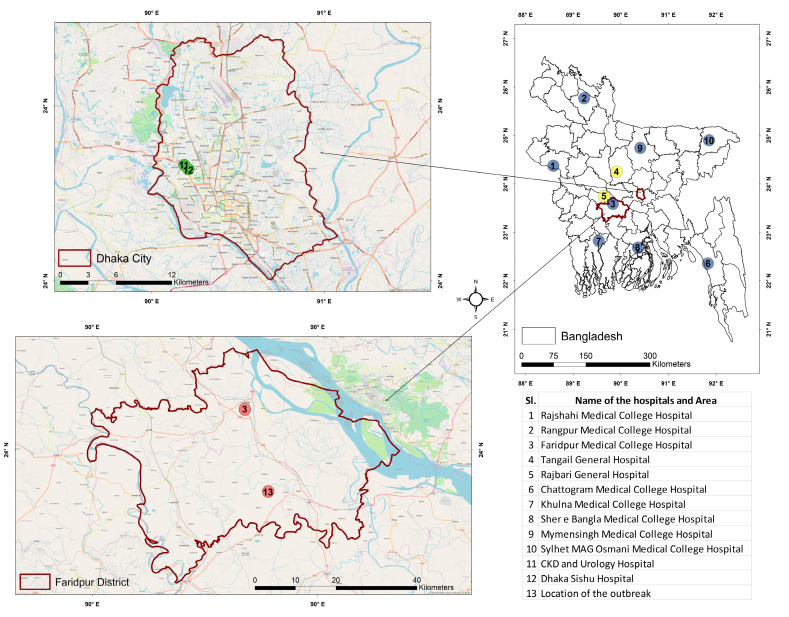
Map of Bangladesh, showing the locations of sentinel hospital-based surveillance sites (1–10); among them, two are passive surveillance sites (4, 5); healthcare facilities in Dhaka district, where confirmed Nipah cases were admitted (11–12) and the location of the outbreak at Faridpur district (Location of the household of “Case X”, “Case Y” and “Case Z”) (13).

The sentinel hospital-based surveillance component is being executed through ten tertiary care hospitals in Bangladesh. Eight of these healthcare facilities are located in eight strategic divisions of the country where the surveillance personnel actively look for suspected Nipah cases among the admitted patients of the Medicine and Pediatrics department (Table 1, Figure 1). The two remaining hospitals are located in two chosen districts where a large number of Nipah cases were identified in previous years (Figure 1).

#### 2.1.2. Event-Based Surveillance

In addition, event-based surveillance is run by IEDCR, where information on atypical or suspected Nipah cases is reported from healthcare facilities other than the sentinel surveillance sites. The information is scrutinized and verified upon receiving such notification, as per the suspected Nipah case definition (Table 1).

### 2.2. Nipah Case and Cluster Identification

On 20 January 2020, Dhaka Shishu Hospital notified the surveillance authority through the event-based platform of a female patient aged below five years, “Case Y”, suffering from atypical and rapidly progressing encephalitis and non-responsive to conventional treatment. Moreover, it was reported that the mother of “Case Y”, designated as “Case X”, was also suffering from the similar illness, and had been admitted to the Kidney Disease Center (CKD) and Urology Hospital, Dhaka (Figure 1). An investigation team documented the clinical findings, collected blood and throat swab samples, and communicated with respective hospital authorities regarding the isolation of patients and maintenance of health safety measures by the health care providers during patient handling. The exposure history of both cases was explored to identify the mode of virus transmission and the potential for human-to-human transmission. Meanwhile, to identify more cases of this encephalitis cluster, caregivers of the hospitalized suspected cases were enquired about their physical condition and other sick persons in their communities who had been suffering from or recently died with similar symptoms (Table 1). Initial inquiry revealed that both “Case X” and “Case Y” consumed raw date palm sap (DPS) a week prior to symptom onset, and “Case Z”, a relative of the disease-stricken family, died of similar symptoms two days back. The history of possible exposure and clustering pattern of the cases led to a strong suspicion of NiV infection. Therefore, the specimen of “Case X” and “Case Y” were sent to the virology laboratory at IEDCR and icddr,b (reference laboratories for the confirmation of NiV infection in Bangladesh) on the same day for the NiV confirmatory tests. Serology and RT-PCR were conducted, and both cases were positive for human NiV infection.

### 2.3. Nipah Outbreak Investigation

Upon confirmation of NiV infection, strict isolation and other infection prevention and control (IPC) measures were put in place at the hospitals where both cases were admitted. An outbreak investigation was launched to identify and test close contacts for NiV infection, to determine the exact source of infection, and to ensure complete documentation of clinical progression and events from the disease onset. Exploration was done in the affected community to find and investigate additional cases, including the deceased relative “Case Z”, who suffered from similar symptoms before passing away. Simultaneously, clinical and laboratory follow-ups were conducted for the two confirmed cases admitted at the hospital. Although the mother “Case X” survived the acute phase of infection, her daughter “Case Y” unfortunately passed away. Thirty days since the confirmation of infection, “Case X” was discharged from the hospital upon testing negative for RT-PCR for the NiV on two consecutive occasions.

### 2.4. Routine Follow-Up

A systematic routine follow-up of “Case X” was conducted through the surveillance platform after discharge from the hospital to document the NiV infection sequelae and detect any relapse or recrudescence. Over-the-phone follow-up was conducted at every 3-month interval, and thorough follow-up was ensured at every 6-month interval. The rigorous follow-up included clinical evaluation of existing health problems by physicians with expertise in neurology, psychiatry, and ophthalmology. In addition, essential investigations and assessments of the immune response against NiV infection were conducted. Thorough documentation of physical and psychological sequelae is conducted during the in-person follow-up.

### 2.5. Follow-Up after Conception and Childbirth

During a routine follow-up of the survivor (“Case X”), the surveillance authority was informed of the conception. As per the Nipah surveillance protocol; routine follow-up was continued and no specimen collection or laboratory investigation was done. Upon reaching term, the survivor was advised by her gynecologist to undergo a caesarian section for child delivery. As this person suffered from NiV infection and was the first reported NiV infection survivor to give birth, extra precautions were taken by the respective hospital authority. A healthy baby boy was delivered at Bangabandhu Sheikh Mujib Medical College Hospital (BSMMCH), one of the sentinel surveillance hospitals. 

Since birth, the surveillance physician with expertise in pediatric and neonatal diseases thoroughly evaluated the child. The survivor was also under follow-up while she recovered from the procedure. Being the baby of a NiV infection survivor, to exclude the possibility of vertical transmission of NiV infection, the newborn was tested for the presence of the NiV and NiV-specific immune response. Three milliliters of blood were collected from the infant, which was later processed on-site and sent to the reference laboratories at Dhaka for evaluation. Anti-Nipah IgM, IgG, and RT-PCR were performed on the specimen. Three days after delivery, the survivor and the newborn were discharged.

### 2.6. Laboratory Methodology

The samples were tested at the virology laboratory of IEDCR and icddr,b. At IEDCR, ELISA for Anti NiV IgG and IgM were conducted to observe the humoral immune response. The in-house assay was developed as per the methodology recommended by CDC, Atlanta [6,10,17]. For detection of anti-Nipah IgM in human sera, a microtiter plate (Corning^®^, Glendale, AZ, USA) was coated with anti-human IgM antibody (1:500, KPL Antibodies & Conjugates (Seracare, Milford, MA, USA) in Phosphate-Buffered Saline (PBS) (Sigma-Aldrich, St. Louis, MO, USA) at pH 7.4 overnight at 4 °C. Following wash, pretreated patient serum (heat inactivated and chemically treated) was added to the appropriate wells of the rows A to D of the microtiter plate at four different dilutions (1: 100, 400, 1600 & 6400) and similarly the same sample was added to the rows E to H of the same microtiter plate and kept at 37 °C for an hour. After incubation, Nipah cell slurry (inactivated NiV culture with Vero E6 cell line, prepared and provided by CDC, Atlanta) mixed with normal human sera (provided by CDC, Atlanta) was added to the wells of the rows A–D and slurry of control Vero E6 cell line was added to the remaining rows E–H and was incubated at 37 °C. Hyper-immune mouse ascitic fluid (HMAF, an ascitic fluid obtained from mouse which was immunized by NiV) consisting of Nipah antibody (dilution 1:4000, provided by CDC, Atlanta) was then added to the plate and incubated. HRP conjugated anti-mouse IgG, IgM (Thermo Fisher Scientific, Waltham, MA, USA) at a dilution of 1:8000 was added. 2,2′-azino-di (3-ethylbenzthiazoline-6-sulfonate) (ABTS) (Seracare, Milford, MA, USA) was used as the substrate and incubated for 30 min. Optical density (OD) of each well was measured at 414 nm using Epoch2 microplate reader (BioTek, Santa Clara, CA, USA). After every step the plate is incubated at 37 °C for an hour followed by washing three times with washing buffer (PBS, and 0.1% Tween20) (Sigma-Aldrich, St. Louis, MO, USA).

For the detection of IgG antibodies, microtiter plate (Corning®, Glendale, AZ, USA) was coated with 1:1000 diluted Nipah cell lysates of NiV culture with the Vero E6 cell line (prepared and provided by CDC, Atlanta) in PBS and incubated overnight at 4 °C for positive portion (wells of the rows A–D) and the for the assay controls, the plate was coated with Vero e6 cell line (wells of the rows E–H). Following wash with PBS-Tween, pretreated patient serum (heat inactivated and chemically treated) was added to the plate using the same dilutions as mentioned above and kept at 37 °C for an hour. HRP conjugated mouse anti-human IgG (Accurate Chemical & Scientific Corporation, Westbury Ave, Carle Place, NY, USA) at a concentration of 1:4000 was added after incubation to the wells and ABTS was used as the substrate. The color development and OD measuring process was the same as IgM detection. The result was calculated by subtracting the OD value from the Nipah cell slurry (rows A–D) by the corresponding OD values of assay control, E6 cell slurry (E–H) and the result was interpreted by the sum of derived OD values (positive if >0.45 for IgM and >0.95 for IgG). Serum diluent {5% skim milk) Difco/vwr, Radnor, PA, USA) in 0.1% Tween 20 in PBS} was used as negative control and, were used as ELISA positive controls.

Viral nucleic acid was extracted from 200 μL of serum or swabsamples collected in lysis buffer (NucliSENS® easyMag, bioMerieux Inc., Rodolphe St., Durham, NC, USA) using InviMag Virus DNA/RNA Mini Kit (INVITEK Molecular, Berlin-Buch GmbH, Germany) on Kingfisher Flex 96 (Thermo Fisher Scientific Inc., Waltham, MA, USA) automated nucleic acid extraction system according to the manufacturer’s instructions. The nucleic acids were eluted in 120 μL of elution buffer and stored at −80  °C. Five microlitres of extracted nucleic acid was used as a template for 25 µL of one step RT-PCR rection volume. Initially, *Taq*Man PCR assay was used to screen NiV RNA using NiV N gene-specific primers and probe, as described by Lo et al. [6]. One-step RT-PCR reactions were performed using the AgPath-ID one-step RT-PCR kit (Applied Biosystems, Foster City, CA, USA) as described previously by Lo et al. [6]. In brief, reverse transcription was carried out for 10 min at 50 °C followed by initial denaturation at 95 °C for 3 min, and PCR was conducted for 45 cycles at 95 °C for 15 s and 60 °C for 1 minute. The results were analyzed using CFX Maestro Software 1.1 version (Bio-Rad Laboratories, Inc., Hercules, CA, USA) for quality of the amplification curve and determination of cycle threshold (Ct) values. The samples was considered positive if Ct ≤ 37 and of good amplification curve.

### 2.7. Statistical Analysis

We used descriptive statistics to characterize the demographic and clinical characteristics of the Nipah case patients, their exposure history, the findings from NiV infection survivors, and the newborn follow-up. The categorical laboratory findings were described on the nominal scale, and the quantitative observations were expressed on the interval scale. No additional statistical tools were exercised.

### 2.8. Ethical Consideration

Informed written consent was obtained from the suspected Nipah cases or their legal guardian, as well as from the individuals enrolled in the study, during the implementation period. Informed assent was obtained from “Case Y”, as well as written permission from the legal guardian. Concerning the newborn, informed assent was obtained from the legal guardian. This study protocol (PR-2005-026) was reviewed, and ethical approval was obtained from the ethical review committee of icddr,b.

## 3. Results

### 3.1. Findings from Outbreak Investigation

**Figure 2:**
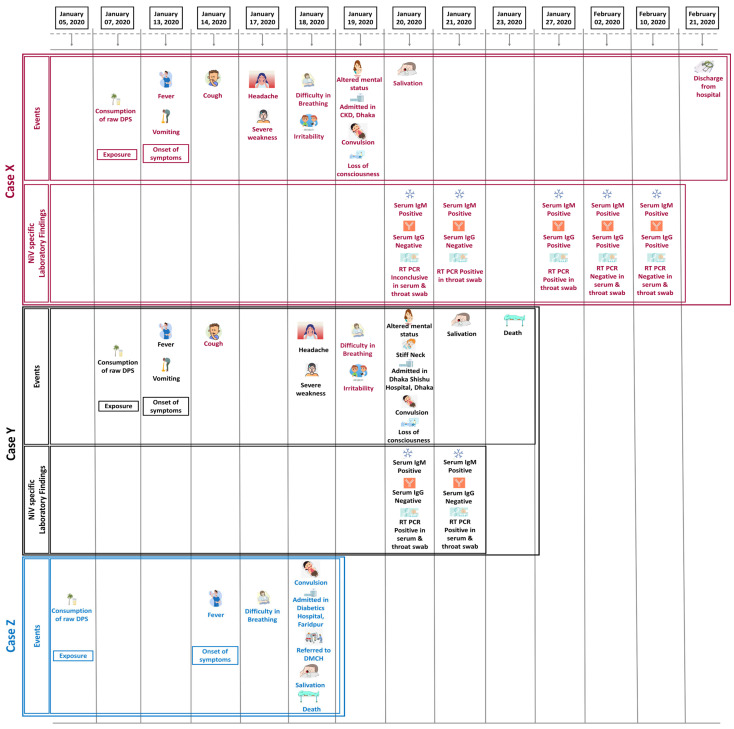
Findings from clinical and laboratory investigation according to the timeline.

We identified one cluster consisting of Two confirmed cases (“Case X” and “Case Y”) and one probable case (“Case Z”) of NiV infection. The two confirmed cases were females aged 30 and 4 years 6 months, and the probable case was a 25-year-old male. “Case Y” was the daughter of “Case X”. “Case Z” was a relative who lived in an adjacent household in a village of Nagarkanda upazilla, located in the Faridpur district (Figure 1). The investigation revealed that all three cases had a history of raw date palm sap consumption before their onset of symptoms, and no other epidemiological link was present (Figure 2). “Case X” and “Case Y” consumed raw DPS from the same source, and “Case Z” consumed from a different source.

The onset of symptoms for all three cases was during the second week of January 2020, and all had a history of consuming raw date palm sap (DPS) in the first week of the same month (Figure 2). The median duration from exposure to the onset of illness was six days. In every case, the illness began with a fever, rapidly progressing to difficulty breathing and convulsion. The probable case (“Case Z”) was admitted to a private hospital in Faridpur as his symptoms worsened. He was referred to Dhaka Medical College Hospital (DMCH) for better treatment but, unfortunately, passed away on the way to Dhaka. Both the confirmed cases suffered from illness, similar to “Case Z”, and were directly brought to Dhaka as their conditions deteriorated. They were reported a day after being brought to Dhaka, and specimens were tested to confirm infection. The “Case X” and “Case Y” both had anti-Nipah IgM (in serum) on the first day of detection (7 days after onset of symptoms). “Case Y” was RT-PCR positive for NiV on the same day (in throat swab and serum), but the result was inconclusive for “Case X”. “Case Y” lost consciousness on the day of hospitalization, and just two days later, she succumbed to the infection. RT-PCR test for NiV were positive for “Case X” a day after “Case Y”. Despite being in critical condition, she survived the initial period of infection and gradually recovered. She developed anti-Nipah IgG, 14 days after the onset of symptoms but was still positive on RT-PCR (in throat swab), implying the potential for human-to-human transmission. Case X first tested negative for NiV RT-PCR on the 21st day of symptom onset and was discharged on 21 February 2020 (39 days since she got sick with NiV infection) upon testing negative for NiV RT-PCR (in throat swab) on two consecutive occasions (Figure 2).

Individuals who came in close contact with all three cases or their bodily secretions were documented and enrolled to exclude person-to-person infection. Fortunately, no secondary case was found.

### 3.2. Survivor Follow-Up

“Case X” survived but had significant neurological complications. As per protocol, she was followed up eight times (four in-person follow up and four follow up over the phone), up to two years since discharge. During the initial six months, she experienced weakness and breathing difficulty on exertion, vertigo, and difficulty in balance while walking. She complained of headaches on reading or remembering past events and had significant difficulty memorizing information. The relatives pointed out that, she had a change in personality since the infection and became less communicative. The psychiatric evaluation suggested that she was suffering from a major depressive disorder. Upon laboratory evaluation, she had persistent anti-Nipah IgG (Table 2). Nine months after recovery from NiV infection, she started receiving psychiatric treatment under the supervision of a psychiatrist.

Over the next six months, she showed gradual physical and psychological improvement. She had one more in-person follow-up in June 2021 and underwent another thorough/extensive neurological and psychiatric evaluation. Despite the persistence of most of the neurological sequelae, all were reduced in severity and intensity. Her psychiatrist also confirmed that she was responding to medication and was recovering well from her depressive illness.

### 3.3. Conception and Childbirth

The survivor conceived in November 2021 and was under routine obstetric follow-up. Her pregnancy was uneventful except for occasional weakness and difficulty sleeping. Remarkably, symptoms associated with the neurological sequelae of NiV infection gradually worsened as she approached term. She was admitted to BSMMCH, Faridpur, on 11 August 2022, and on the same day, she underwent caesarian section. A healthy baby boy was born at 40 weeks of gestation. Immediately after birth, the neonate scored the highest on the APGAR evaluation (a test to evaluate the health of newborns) and showed no deformity or physical disability on his initial physical examination. Unfortunately, 2 days after birth, the child developed jaundice. Several phototherapy sessions were provided, and upon significant improvement, the mother and her baby were discharged five days after childbirth.

### 3.4. Evidence of Humoral Immunity against NiV Infection

Parameters of humoral immunity of the survivor against NiV was closely monitored during and after the active infection. Anti Nipah IgM was detected on 7th day of symptom onset and the titre peaked till 8th day which was followed by a steady decline. After fourteen days of disease onset, serum IgG against NiV was first detected which peaked over the next ten months. The last recorded anti NiV IgG of Case X showed a declining trend. The baby of “Case X” was negative for anti-Nipah IgM and RT-PCR for NiV but positive for anti-Nipah IgG antibody. This finding was consistent with the declining trend of IgG observed from maternal specimen, before conceiving the child (Table 2).

## 4. Discussion

The findings from outbreak investigation, laboratory, clinical assessment, and survivor follow-up suggest that the three cases of the suspected Nipah cluster were infected with the NiV. Among them, two were confirmed via laboratory investigation, and the other died with Nipah-like symptoms with epidemiological evidence of NiV infection; hence designated as a probable Nipah case. The appearance and persistence of anti Nipah IgM for both the confirmed cases are quite similar to other evidences of NiV specific immune response [16]. However, the appearance of anti Nipah IgG was a bit delayed for the survivor in this study, compared to other NiV survivors. This may be related to individual biological factors which require further exploration. The persistence of NiV RNA in a throat swab two weeks after the infection may seem intriguing but, similar evidences exist in cases from India where the survivor kept testing positive in RT-PCR, as long as three weeks since onset of symptoms [15].

The only survivor from this cluster carried immunological evidence of NiV infection, and afterward, her newborn child was also found with evidence of immunity against the NiV. This implies that the immunogenic property had been transferred from the maternal circulation to the newborn.

The placenta acts as a bridge between a mother and her unborn child, through which maternal properties are shared with the offspring. One of the crucial materials shared between maternal and fetal circulation is the pathogen-specific immune properties that occur throughout pregnancy and, on most occasions, are followed postnatally through breast milk [18,19]. We documented the presence of anti-Nipah IgG in the circulation of a child born to an individual with a history of confirmed NiV infection, suggesting a trans-placental transfer of pathogen-specific immune properties. The preliminary evidence is consistent with similar events involving other viruses and demands a further thorough assessment of NiV-specific immune properties in the newborn. In addition, serial antibody titre assessment should be conducted for the mother and child to observe, compare and contrast the level of immune properties between maternal and neonatal immune systems.

Infants and newborns are especially at risk of viral infections as their immune systems are still developing [19]. During this vulnerable period, susceptibility to infection can be minimized by passive immunity obtained from maternal circulation [20,21]. Albrecht et al. described that passive protection through immune transfer from the mother protects from diseases such as pertussis and influenza A for nearly six months [21]. Furthermore, if infected, maternal antibodies in the newborn reduce the likelihood of developing the severe disease [22,23]. Evidence from this study further demands the assessment of neutralizing potential of the anti-Nipah antibody present in the newborn’s circulation and its persistence over time. An actual neutralization test against the NiV demands a BSL-4 facility, so a pseudo-virus neutralization should be the next course of action.

Available scientific evidence suggests that immunization of pregnant women stimulates the pathogen-specific antibody concentration and results in high cord blood antibody concentration; hence, it is a reliable measure to prevent disease in both her and her offspring [22,24]. Similar to natural maternal infection, this is also associated with a reduced risk of post-birth infection [19]. In light of the ongoing COVID-19 pandemic and considering the similar potential of the NiV, the drive for effective vaccine development has been reinforced. Once a vaccine is developed, evidence from this study will assist compare the anti-Nipah immunological features of infants born to vaccinated and naturally infected mothers.

## 5. Conclusions

To our knowledge, this study is the first to investigate and report the vertical transfer of NiV-specific immune properties. It warrants further exploration of its effectiveness in virus neutralization and its potential to protect newborns. This will also be a reference for vaccine recommendations for pregnant/young women against the NiV.

## Figures and Tables

**Table 1:** Surveillance case definitions of human NiV infection and cluster.

Type of Case/Event	Criteria
Suspected Nipah encephalitis	Fever or history of fever (axillary temperature > 38.5 °C) **AND** evidence of acute brain pathology (e.g., altered mental status, new-onset seizures, or new neurological deficit either diffuse or localized to the brain)
Suspected Nipah pneumonitis	The onset of illness within the last seven days AND fever or history of fever (axillary temperature > 38.5 °C) **AND** severe shortness of breath (i.e., dyspnea that prevents the patient from walking unassisted for not more than ten steps) **AND** chest radiograph consistent with diffuse acute respiratory distress syndrome
Probable Nipah infection	History of Nipah encephalitis or pneumonitis AND established epidemiological link with at least one confirmed Nipah case but absence of laboratory confirmation due to the patient’s death
Confirmed Nipah infection	Laboratory confirmation of the infection (identification of anti-Nipah IgM OR Viral particles through RT-PCR) with or without a history of Nipah encephalitis/pneumonitis
Suspected Nipah cluster	Two or more suspected Nipah case-patients residing within 30 min walking distance from each other who had onset of similar illnesses within three weeks of one another or had epidemiologic linkages to one another

**Table 2:** Serum anti-Nipah IgM and IgG of “Case X” and her newborn baby.

Specimen Collection Date	Subject	Sum of Derived OD Values for IgM	Result Interpretation	Sum of Derived OD Values for IgG	Result Interpretation
20 January 2020	Case X	4.867	Positive	0.187	Negative
21 January 2020	Case X	8.020	Positive	0.443	Negative
27 January 2020	Case X	6.159	Positive	7.839	Positive
2 February 2020	Case X	5.080	Positive	8.340	Positive
10 February 2020	Case X	3.848	Positive	8.883	Positive
5 November 2020	Case X	-	-	11.715	Positive
1 June 2021	Case X	-	-	9.760	Positive
16 August 2022	Baby of Case X	0.147	Negative	7.514	Positive

Note: “-” = Not done.

## Data Availability

The datasets used and/or analyzed during the current study are available from the corresponding author upon reasonable request. Please contact the author for data requests.
